# A meta-review of systematic reviews and meta-analyses on outcomes of psychosocial interventions in heart failure

**DOI:** 10.3389/fpsyt.2023.1095665

**Published:** 2023-03-10

**Authors:** Catarina Nahlén Bose

**Affiliations:** Department of Health Sciences, The Swedish Red Cross University, Huddinge, Sweden

**Keywords:** anxiety, depression, heart failure, intervention, psychosocial, quality of life, review

## Abstract

**Introduction:**

Chronic heart failure is a severe condition that influences not just the physical dimension but also the mental dimension in patients. Comorbidity of depression and anxiety are prevalent and the quality of life is reduced. Despite the psychological impact there are no recommendations in the guidelines for psychosocial interventions for people with heart failure. The aim of this meta-review is to synthesize results of systematic reviews and meta-analyses on the outcomes of psychosocial interventions in heart failure.

**Methods:**

Searches were conducted in PubMed, PsychInfo, Cinahl and the Cochrane Library. In total, seven articles were included after screening 259 studies for eligibility.

**Results:**

The included reviews had, in total, 67 original studies included. The measured outcomes in the systematic reviews and meta-analyses were; depression, anxiety, quality of life, hospitalization, mortality, self-care and physical capacity. The results are inconsistent but show some short-term benefit of psychosocial interventions for reduced depression and anxiety and improved quality of life. However, the long-term effects were sparsely followed up.

**Discussion:**

This meta-review appears to be the first in the field of the efficacy of psychosocial interventions in chronic heart failure. This meta-review identifies gaps in the current available evidence that need to be further explored, such as booster sessions, longer follow-up time for evaluation and incorporating clinical outcomes and measures of stress processes.

## Introduction

The prognosis of chronic heart failure (CHF) is serious, as the survival rate is comparable with common forms of cancer (1). The prevalence of CHF is estimated to be 1–2 percent of the population but increases sharply with age, where the prevalence in people older than 70 years is >10 percent. Heart failure is a clinical syndrome with symptoms such as breathlessness, fatigue and ankle swelling and may have objective signs such as pulmonary crackles. CHF is caused by functional and/or structural pathology and the outcome may be a reduced cardiac output or increased intracardiac pressure (2). Living with heart failure affects several dimensions of the person’s life, not just the physical but also their emotional, social and spiritual dimensions. CHF requires people to adjust to a new life situation and adopt coping strategies (3). The prevalence of depression and anxiety is high (4, 5) and depression is an independent predictor of mortality in CHF (6). Moreover, the quality of life (QoL) is reduced where depression has been found to correlate with QoL (7). With regard to the psychological impact, patients with CHF may, besides pharmacological and device treatment, need psychosocial interventions. Yet, the latest guidelines for CHF are lacking recommendations for psychosocial interventions (2), most likely because the evidence is not coherent or sufficient. In the guidelines for CHF, level A evidence is data generated from multiple randomized controlled trials or meta-analyses (2). This meta-review aims to synthesize results of systematic reviews and meta-analyses on outcomes of psychosocial interventions in heart failure.

## Methods

### Eligibility criteria

The inclusion criteria were systematic reviews and meta-analyses on psychosocial interventions for persons with heart failure that evaluate psychological outcomes. Psychosocial interventions were defined as interventions that had a psychoeducative component, e.g., cognitive behavioral therapy (CBT) or coping skills training. The studies should have been published within the last 10 years.

Exclusion criteria were: original studies not written in English, comparative reviews or meta-analyses between different treatments for depression, e.g., between pharmacological treatment and psychosocial interventions, reviews or meta-analyses on cardiac rehabilitation or interventions solely focusing on tai-chi, yoga or mindfulness or other interventions that lack a psychoeducative component and reviews with mixed patient populations.

### Search strategy and quality assessment

Searches in the following databases were performed in September 2022: PubMed, PsychInfo, Cinahl and Cochrane library. Reference lists in the articles, that were read in full text, were also screened for eligible studies. The search string was “(((heart failure AND (intervention OR therapy)) AND (psycho* OR coping)) AND (review OR meta-analys*).” The filter was set to article-type: Meta-analysis, Review, Systematic review in PubMed and Literature review, Systematic review, Meta-analysis in PsychInfo.

AMSTAR-2 was used as a guide for the quality assessment of the systematic reviews and meta-analyses (8). The AMSTAR-2 tool consists of 16 quality appraisal items. Based on the evaluation a recommended level of critically low to high quality was suggested. The tool does not generate a quality score. Seven of the items are considered critical, for example, risk-of-bias assessment in the individual studies. If the study did not fulfill one critical item, the recommended level is low quality and if two critical items are not fulfilled the study is assessed as critically low quality.

### Data extraction and analysis

Data from the articles concerning type of review, numbers and types of studies included, total number of participants, intervention, comparator, outcomes and effect size were extracted to an article matrix. Furthermore, all original studies included in the systematic reviews and meta-analyses were charted in a table to investigate how many times the individual original studies were included in the systematic reviews and meta-analyses.

The results of the studies were grouped based on the outcome measures and described in a narrative form.

## Results

### Study selection and characteristics of included studies

**Figure 1:**
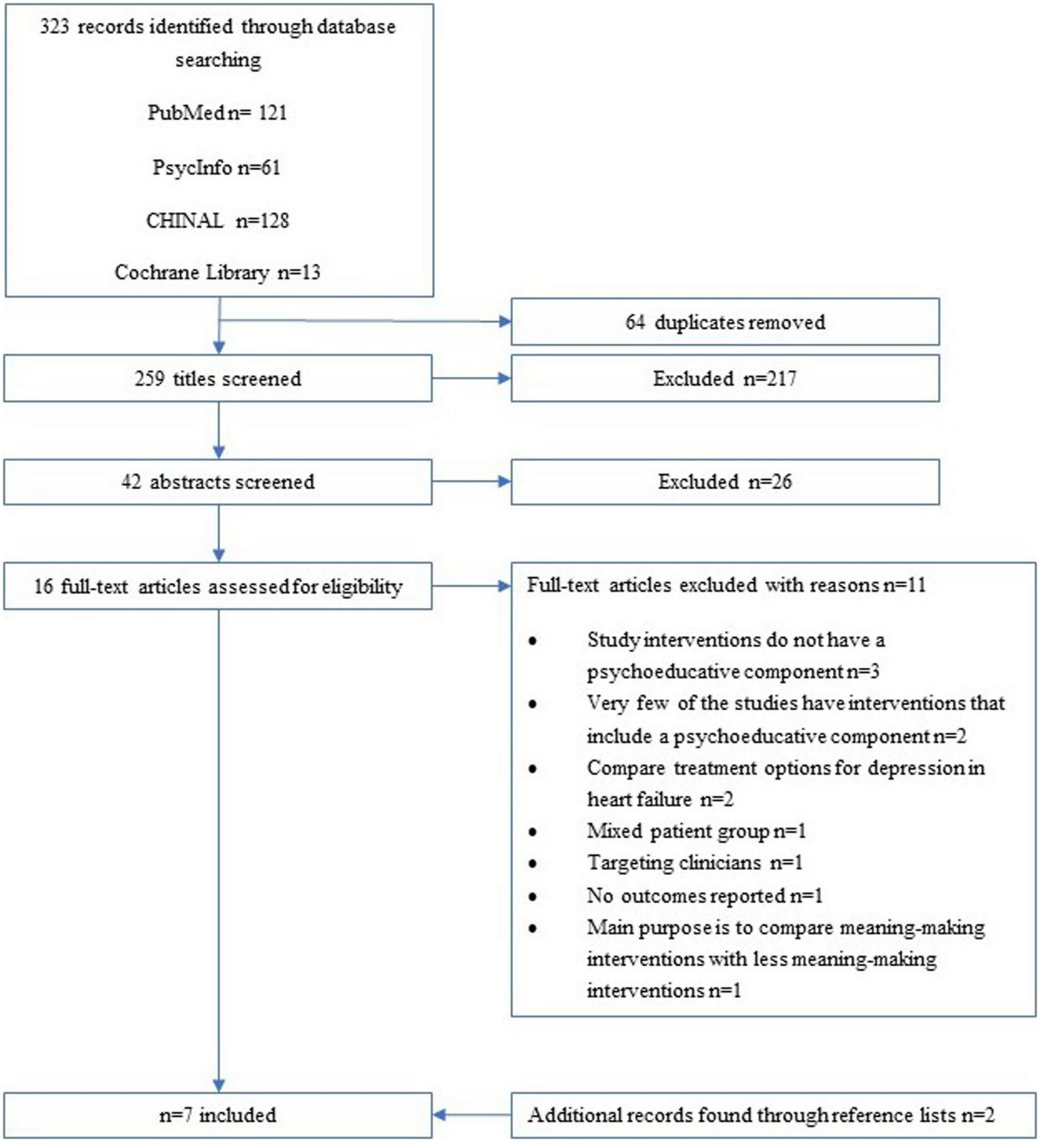
Flowchart of identification, screening and inclusion process.

Initially, 259 titles were screened after duplicates had been removed. After abstracts were screened, 16 articles were read in full text. Eleven of those articles did not meet the eligibility criteria and the reasons for exclusions of each article can be found in Supplementary material 1. An additional two articles were found to meet the eligibility criteria through reference screening of the full text articles, hence seven articles were included in the study (9–15) (Figure 1).

The characteristics of the included studies are shown in Table 1. Of the seven studies, four included only randomized controlled trials and three included a mixture of both randomized controlled trials and non-randomized controlled trials. Six of the studies had performed meta-analyses. Two of the studies focused solely on CBT and the rest had a mixture of CBT and other forms of psychosocial interventions like coping skills training. The main comparator was usual care solely or usual care and/or heart failure education. In total, 67 individual original studies were included in the systematic reviews and meta-analyses, where 19 of the original studies were included in several (between 2 and 7) of the systematic reviews and meta-analyses (Supplementary material 2). Possible explanations for the variability of the how many times an original study was included in the reviews could be the intervention type or sample size, e.g., if it was a pilot study or a full-scale study. The different outcome measures in the systematic reviews and meta-analyses were depression, anxiety, quality of life (QoL), hospitalization, mortality, self-care and physical capacity. The most frequent measured outcomes were depression and QoL, that all reviews had included as outcomes. The other outcomes were included on average in two to three of the reviews.

**Table 1:** Article matrix.

First author and year published	Type of review	Number of studies included and design of studies included	Total number of participants	Intervention	Comparator	Outcomes	Effect size, SMD (95% CI)
Chernoff et al., 2022	Systematic review and meta-analysis	23 RCTs except 1 that had an incomplete randomization (15 included in the meta-analysis)	1,370 included in the meta-analyses	Psychosocial interventions – two groups: CBT and stress management	Mostly usual care and/or heart failure education	Depression Anxiety QoL Hospitalization Mortality	Post-intervention (all studies CBT + stress management) Depression −0.41 (−0.66 to −0.17) *p* = 0.001, *k* = 15 Anxiety −0.33 (0,51 to −0.15) *p* < 0.001, *k* = 8 QoL 0.14 (−0.002 to 0.29) *p* = 0.053, *k* = 8
Gathright et al., 2021	Systematic review and meta-analysis	23 RCTs	2,294	Stress management interventions defined as approaches to strengthen an individual’s skills to identify, understand, and cope with psychological and physical stress.	Mostly usual care and/or heart failure education	Depression Anxiety QoL Exercise capacity	*First post-intervention assessment:* Depression 0.39 (0.03–0.75), *k* = 13 Anxiety 0.49 (0.09–0.89), *k* = 10 QoL 0.82 (0.40–1.24), *k* = 16 Exercise capacity 0.57 (0.20–0.95), *k* = 14 The last assessment post-intervention did not show any significant differences between intervention and control groups
Helal et al., 2020	Systematic review	9, 5 RCTs and 4 NRSI	757	Psychological interventions. Mainly CBT and coping skills training	Mostly usual care and/or heart failure education	Depression QoL Hospitalization Mortality	N/A
Jeyanantham et al., 2017	Systematic review and meta-analysis	6, 5 RCTs and 1 NRSI (observational)	320	CBT	Mostly usual care	Depression QoL Hospitalization Mortality	*Post-intervention* Depression −0.34 (−0.60 to −0.08) *p* = 0.01, *k* = 5 *3 months FU* −0.32 (−0.59 to −0.04) *p* = 0.03, *k* = 5 *Post intervention* QoL −0.31 (−0.58 to −0.05) *p* = 0.02, *k* = 5 No difference in QoL after 3 months. No differences in hospitalization or mortality
Jiang et al., 2018	Systematic review and meta-analysis	25 RCTs (in 29 articles)	3,837	Psychological interventions defined as interventions based on psychological principles, such as CBT, motivational interviewing, nondirective counseling, and supportive therapy	Usual care	Self-care QoL Physical function	No effect size on self-care as high heterogeneity recommended not to combine results. Anxiety (short-term FU) −0.07 (−0.59 to 0.45), *k* = 4 (mid-term FU) −0.69 (−1.69 to 0.31), *k* = 4 (long-term FU) 0.04 (−0.45 to 1.25), *k* = 2 QoL (3-months FU) combined MD −7.53 (−12.83 to −2.23), *k* = 3 Not significant at 5–6 months FU. Physical function (6-months FU) combined MD 30.17 (−13.85 to 74.19) *p* = 0.18, *k* = 3
Peng et al., 2019	Systematic review and meta-analysis	8 RCTs	480	CBT	Mostly usual care and /or heart failure education	Depression QoL Self-care 6-min walk test distance	Depression −0.27 (−0.47 to −0.06) *p* = 0.01, *k* = 5 QoL 0.21 (−0.01 to 0.42) *p* = 0.06, *k* = 5 Self-care 0.12 (−0.18 to 0.42) *p* = 0.44, *k* = 2 6-min walking test 0 (−0.28 to 0.28) *p* = 0.99, *k* = 3
Samartzis et al., 2013	Meta-analysis	16 RCTs	1,074	Psychosocial interventions defined as a structured nonpharmacologic intervention conducted by health professionals that is focused on improving the psychologic and/or social aspects of a patient’s health	Usual care	QoL Depression	QoL 0.46 (0.19–0.72) *p* < 0.001, *k* = 16 Depression 0.98 (0.01–1.94), *k* = 3

RCT, Randomized Controlled Trial; NRSI, non-randomized studies of interventions; CBT, Cognitive behavioral therapy; QoL, Quality of Life; SMD, Standardized Mean Difference; CI, Confidence Interval; FU, Follow-up; MD, Mean Difference; *k* denotes number of studies included in the meta-analysis.

The quality appraisal with AMSTAR-2 yielded five studies to have moderate quality (9–13), one study to have low quality (14) and one to be of critically low quality (15). The article with critically low quality had, for instance, not performed a risk-of-bias assessment of the individual articles which is consider a critical item in AMSTAR-2.

### Depression

All seven reviews had depression as an outcome measure post-intervention, where two of the reviews also reported longer-term assessments (10, 12). Two of the reviews included patients with CHF and comorbid depression or depressive symptoms (11, 12). In the five meta-analyses post-intervention, four meta-analyses reported statistically significant reduction in depression with a small to moderate effect size (0.27–0.41) (9, 10, 12, 14). One meta-analysis reported a non-significant result, however, the authors mentioned results that showed a trend for reduction in depression in the intervention group (15). One meta-analyses also divided the meta-analyses for CBT and stress management intervention where both showed significant reductions in depression (CBT −0.37, 95% CI −0.70 to −0.05, *p* = 0.024, Stress management −0.51, 95% CI −0.83 to −0.19, *p* = 0.002) (9). Follow-up assessments showed inconsistent results where one meta-analysis had sustained reductions in depression after 3 months with a moderate effect size (12) and one meta-analysis could not show a sustained effect at last follow-up assessment (21.86 weeks ±14.65, range = 12–52 weeks) (10).

Two studies had not performed a meta-analysis due to high heterogeneity (11, 13) and presented the result in a narrative form. In the systematic review by Jiang et al. (13) 10 studies evaluated depression, and four reported significant reduction in depression. Helal et al. (11) divided the synthesis for depression in three groups: (1) CBT: Two of five studies reported statistically significant reduction in depression in the intervention group. The other three studies reported non-significant reductions in depression for the intervention group. (2) Combined CBT and exercise: One study showed statistically significant reduction in depression for the intervention group. The other study did not show significant between-group differences. (3) Other psychological interventions: Three studies (coping skills training, mindfulness-based psychoeducation, and innovative holistic meditation) showed statistically significant reduction in depression in favor for intervention group.

### Anxiety

Three of the reviews reported anxiety as an outcome measure (9, 10, 13). Two meta-analyses showed statistically significant improvements in anxiety with a moderate effect size (0.33 and 0.49) (9, 10). In one meta-analysis no significant improvements were found (13). Meta-analyses on follow-up assessments between 3 and 12 months could not find any significant effect on anxiety (10, 13).

### Quality of life

All seven reviews had quality of life as an outcome measure. Six of the reviews performed meta-analyses where four reviews found statistically significant improvements in quality of life with a moderate to high effect size (pooled standardized difference 0.31–0.82) (10, 12, 15) and combined mean difference of −7.53 on the Minnesota Living with heart failure questionnaire (13). The effect was not sustained after 3–6 months follow-up (10, 12, 13). In one of the meta-analyses that did not find a statistically significant effect for all the included studies, divided the studies into CBT and stress management and then found a significant improvement for CBT, with a small effect size (0.20), but not for stress management interventions (9). In the systematic review where no meta-analyses had been performed four (two RCTs, one prospective cohort study and one pilot study) out of seven studies reported statistically significant improvement in HRQoL (11). Furthermore, one meta-analysis found that face-to-face was more effective than telephone interventions (15).

### Clinical outcomes

Three reviews reported clinical outcomes on hospitalizations and mortality (9, 11, 12). In each of the systematic reviews and meta-analyses there were between 1/3 and 2/3 of the included original studies that had data on clinical outcomes. One meta-analysis found no significant effect on mortality or rehospitalizations (12). The two other studies presented the result in a narrative form and the results were inconsistent (9, 11). Three out of five original studies reported less cardiac events in favor of the intervention group in one of the reviews (9). In the other review one out of three original studies reported significant reduction in mortality for the intervention group and all RCTs reported statistically significant reduction in hospitalization rates favoring the intervention group (11).

### Self-care

Two reviews had self-care as an outcome measure (13, 14). One of them had performed a meta-analysis that could not find any significant improvements in self-care (14). The other review did not perform a meta-analysis due to high heterogeneity. Of the nine original studies that evaluated the effectiveness of psychological interventions on self-care, six of the studies reported a positive short-term (at 1–3 months post intervention) effect of psychological intervention on a patient’s self-care behaviors in patients without clinical depression (13).

### Physical capacity

Three reviews had physical capacity as an outcome measure (10, 13, 14). The physical capacity was mainly measured by a 6-min walking test. The findings are inconsistent, where one meta-analysis found significant improvement in physical capacity (10) whereas another meta-analysis did not find significant improvements (14). Longer-term evaluations could not find a significant effect at 3–6 months follow-up (10, 13).

### Moderators

Three of the reviews also performed meta-regression analyses to check for potential moderators of change in the outcome measures (9, 10, 15). The different moderators were; severity of heart failure as measured by New York Heart Association (NYHA) class, mean age, sex, length of study, intervention type, mean ejection fraction (EF) at baseline, proportion using beta-blockers and delivery modality (individual vs. group format, presence of home practice). Female sex was associated with a reduction in depression (9) and anxiety were more successfully reduced when the sample had a higher proportion of females (10). NYHA class I and II were associated with reduction in depression (9) and the effect size of QoL was less when the sample consisted of more patients in NYHA class III and IV (10). With regard to intervention type one review found a difference in QoL where CBT was associated with reduction in QoL whereas stress management interventions were not (9). There was no moderating effect on the other outcomes. One review did not find any significant moderating effect of the variables NYHA class, mean age, sex and length of study on the effect size for QoL (15).

## Discussion

**Figure 2:**
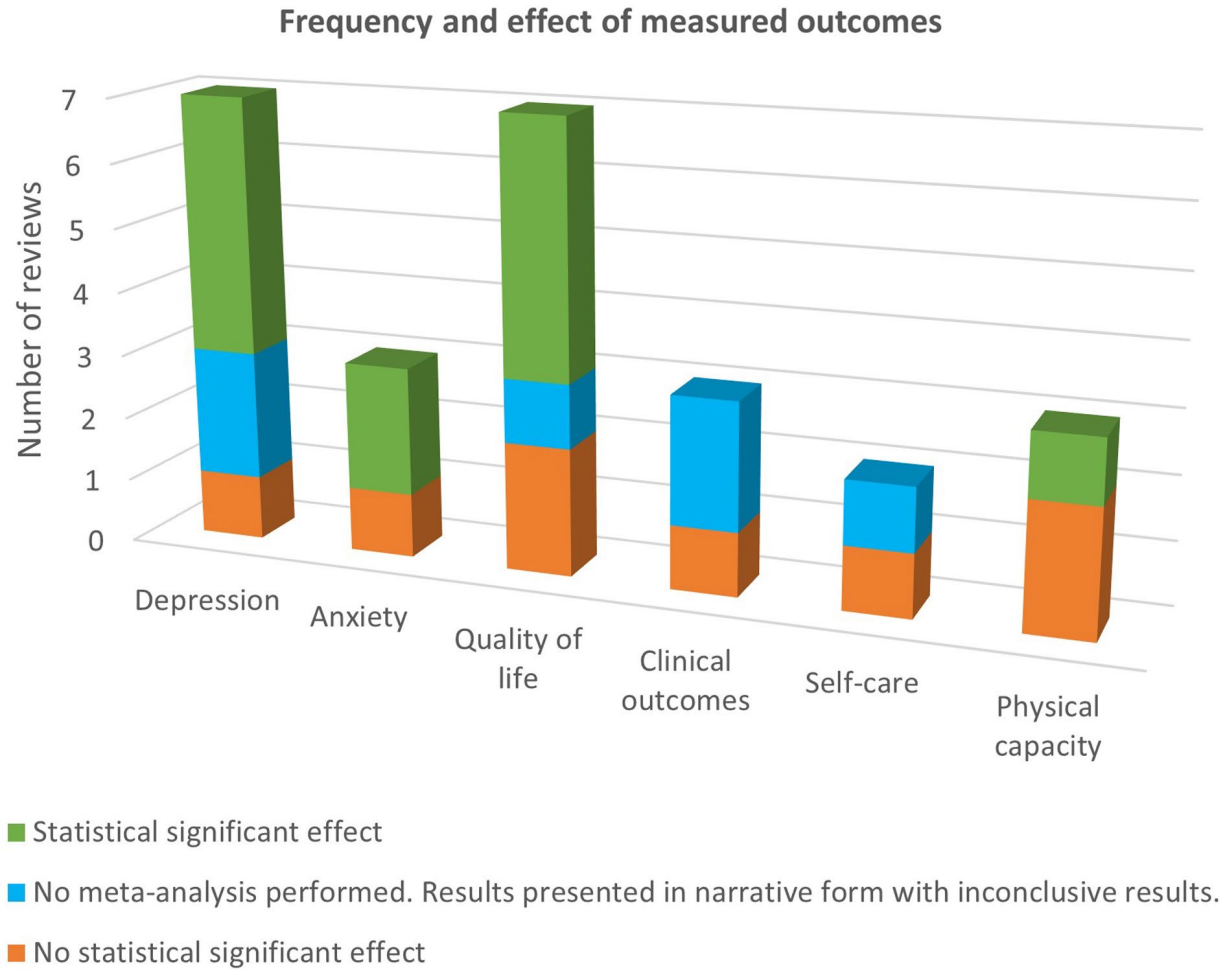
Frequency and effect of measured outcomes at the first post-intervention measurement point.

To the author’s knowledge, this is the first meta-review on the outcomes of psychosocial interventions in CHF. The results were somewhat inconsistent where both positive and negative results were found in mental health, QoL, self-care, clinical outcomes and physical capacity. Figure 2 gives a graphical overview of the results at the first post-intervention measurement point. Four out of five meta-analyses reported a significant reduction in depression with a small to moderate effect size (9, 10, 12, 14). The study that reported a non-significant result, although a trend toward reduction, did not have depression as the primary outcome and included only three original studies in the meta-analysis for depression (15). Anxiety was also reported to be reduced significantly in two of three meta-analyses with a moderate effect size (9, 10). The long-term follow-up assessment for depression and anxiety showed some support for a long-term effect of reduced depression at 3 months but no sustained effect for reduced anxiety (10, 12, 13). The results are somewhat consistent with findings from a meta-analysis on psychosocial interventions in patients with cardiovascular diseases where short-term effects on anxiety and depression was found but was not sustained at follow-up assessments (16). However, there were few studies that had long-term follow-ups which was also the case in the current meta-review. Notably, the meta-analysis that found a sustained effect of reduced depression had a highly selective sample consisting of mostly male with a mean age ranging from 55 to 66 years (12), in contrast to CHF being most prevalent in people over 70 years (2). Furthermore, one meta-analysis in the current meta-review points out that the studies favoring the intervention group had a longer medium durability of the interventions in comparison to studies favoring control (9). This meta-review gives some support to that psychosocial interventions can have a short-term effect on QoL as several of the studies reported significant results (10, 12, 13, 15) and, in a subgroup analysis, for CBT interventions solely (9). The effect was, however, not sustained at follow-up (10, 12, 13). Discussions are raised in several of the included systematic reviews and meta-analysis as to whether booster sessions could promote a long-term effect on the outcome measurements. There could be some support for this suggestion and worthwhile to explore as one meta-analysis that particularly investigated the effect of booster sessions in CBT, albeit with a different patient population, found that interventions with booster sessions were more effective and the effect was more sustainable (17).

Clinical outcomes, as measured by hospitalizations and mortality, were sparsely evaluated and, when it was performed, showed inconsistent results. Besides psychological outcomes and QoL, clinical outcomes are also important factors to consider when evaluating a psychosocial intervention, although it is usually not the primary outcome. Clinical outcomes provide objective measures and could be useful when assessing cost-effectiveness and deciding whether the intervention should be implemented in clinical practice. Sparsely evaluated was also self-care behavior and physical capacity with contradictory results. While self-care behavior is measured by self-assessment, physical capacity is an objective measure. The meta-analysis that found an improvement in physical capacity had also partly included studies with a combination of stress-management and a physical movement component (10).

Although this meta-review identified several included outcome measures for psychosocial interventions, one of the included systematic review and meta-analysis raises the lack of measuring critical stress processes like perceived stress and coping strategies in the original studies (10). Coping strategies have, for instance, been associated with different levels of depression depending on whether adaptive or maladaptive coping was used as a strategy in patients with CHF (18) and therefore could be useful to address and measure.

Another aspect to take into consideration is the format of how the psychosocial intervention is delivered. Patients with CHF might find it straining to go on several visits for reasons such as fatigue. Tele-rehabilitation could be an alternative format of delivery in order to reach more patients who otherwise would decline participation. Tele-rehabilitation interventions in CHF have shown some positive effect on quality of life, physical capacity and mental health (19). Home-based treatment based on self-help is another possible option. Home-based meta-cognitive therapy for cardiac patients have been found to be a feasible approach (20).

## Limitations

This meta-review has some limitations. Firstly, there is no definite consensus on what constitutes a psychosocial intervention. The original studies in the included reviews had different kinds of interventions and it cannot be guaranteed that all of them had a psychoeducative component which was an inclusion criterion in this study. The study cannot conclude which type of psychosocial intervention is favorable for an intended effect. Secondly, there was heterogeneity in the reviews and the original studies often had small sample sizes hence the results should be interpreted with caution. Thirdly, since some of the original studies were included in several of the systematic reviews and meta-analyses, there could have been an overlap in the results. Fourth, the quality according to AMSTAR-2 did not assess any of the articles to be of high quality which might impact the accuracy of the results. Notably, none of the included articles where a Cochrane review. Fifth, this meta-review, although approached in a systematic manner, might not have covered all available data. Finally, this meta-review was performed by one researcher hence there is a risk of bias.

## Conclusion and future direction for research

This appears to be the first meta-review in the field of psychosocial interventions in CHF. The meta-review found that psychosocial interventions in CHF may reduce depression, anxiety and improve quality of life but the results are inconsistent and the support for long-term effects, when measured, were few. Some points are raised to take into consideration for future studies. Interventions should be evaluated with long-term follow-ups and explore whether booster sessions could provide a sustained effect and whether the durability of the intervention has on impact on effect and sustainability. Studies should strive to have adequate sample sizes and include clinical outcomes and measures of stress and coping strategies. Furthermore, large-scale, high-quality studies that compare different types of psychosocial interventions could be useful.

## Data availability statement

The original contributions presented in the study are included in the article/Supplementary material, further inquiries can be directed to the corresponding author.

## Author contributions

The author has contributed to the conception and design of the study, acquisition of data, analysis and interpretation of data, drafting the article and revising it critically for important intellectual content.

## Conflict of interest

The author declares that the research was conducted in the absence of any commercial or financial relationships that could be construed as a potential conflict of interest.

## Publisher’s note

All claims expressed in this article are solely those of the authors and do not necessarily represent those of their affiliated organizations, or those of the publisher, the editors and the reviewers. Any product that may be evaluated in this article, or claim that may be made by its manufacturer, is not guaranteed or endorsed by the publisher.
