# Supplementary material for: A meta-review of systematic reviews and meta-analyses on outcomes of psychosocial interventions in heart failure
Source: Front Psychiatry. 2023 Mar 10;14:1095665. doi: 10.3389/fpsyt.2023.1095665 (PMC10036787; doi:10.3389/fpsyt.2023.1095665)
Supplement: Supplementary file 1 [file Table_1.DOCX]

Supplementary material 1. Excluded articles after full-text reading

| **Article** | **Reasons for excluding after full-text reading** |
| --- | --- |
| Ania-González N, Olano-Lizarraga M, Vázquez-Calatayud M. Interventions to empower cardiorenal patients: A systematic review. *J Adv Nurs.* (2021) 78:363–376. doi: 10.1111/jan.15007 | Two of five studies had a heart failure sample. The other three studies included chronic kidney disease. |
| Cui X, Dong W, Zheng H, Li H. Collaborative care intervention for patients with chronic heart failure: A systematic review and meta-analysis. *Medicine.* (2019) 98:13. doi: 10.1097/MD.0000000000014867 | Focused on collaborative care. Too little information in the article about the interventions in the included studies to assess whether the studies contain a psychoeducative component. |
| Das A, Roy B, Schwarzer G, Silverman M, Ziegler O, Bandyopadhyay D, et al. Comparison of treatment options for depression in heart failure: A network  meta-analysis. *J Psychiatr Res.* (2019) 108:7-23. doi: 10.1016/j.jpsychires.2018.10.007 | Comparison of treatment options for depression in heart failure. Compared exercise, pharmacological antidepressant treatment and cognitive behavioral therapy (CBT). |
| Ischak W, Edwards G, Herrera N, Lin T, Hren K, Peterson M, et al. Depression in Heart Failure: A Systematic Review. *Innov Clin Neurosci*. (2019) 17(4-6):27-38. | Comparison of treatment options for depression in heart failure. Included different types of treatment such as pharmacological, exercise and psychotherapy. |
| Kyriakou M, Middleton N, Ktisti S, Philippou K, Lambrinou E. Supportive Care Interventions to Promote Health-Related Quality of Life in Patients Living With Heart Failure: A Systematic Review and Meta-Analysis. *Heart Lung Circ.* (2020) 29(11):1633-1647. doi: 10.1016/j.hlc.2020.04.019 | Only one of ten included studies had a psychoeducative component (CBT). The other studies concern excercise or education. |
| Li Y, Fang J, Li M, Luo B. Effect of nurse-led hospital-to-home transitional care interventions onmortality and psychosocial  outcomes in adults with heart failure: a  meta-analysis. *Eur J Cardiovasc Nurs.* (2022) 21(4): 307–317. doi: 10.1093/eurjcn/zvab105 | Focused on transitional care interventions and not psychosocial interventions with a psychoeducative component. |
| Olano-Lizarraga M, Wallström S, Martín-Martín J, Wolf A. Interventions on the social dimension of people with chronic heart failure: a systematic review of randomized controlled trials. *Eur J Cardiovasc Nurs.* (2022). doi: 10.1093/eurjcn/zvac051 | Only two of the eight included studies had a psychoeducative component. |
| Oyanguren J, García P, Laguna J, Goya I, Martín S, Lafuente E, et al. Effectiveness and Factors Determining the Success of Management Programs for Patients With Heart Failure: A Systematic Review and Meta-analysis. *Rev Esp Cardiol*. (2016) 69(10):900–914. doi: 10.1016/j.rec.2016.05.012 | Focused on self-care programs, not psychosocial interventions. |
| Sacco S, Leahey T, Park C. Meaning-making and quality of life in heart failure interventions: a systematic review. *Qual Life Res.* (2019) 28:557–565. doi: 10.1007/s11136-018-1993-2 | Main purpose was to compare meaning-making interventions with less meaning-making interventions. The purpose as such was not in line with the current review, that doesn’t aim to compare psychosocial interventions with other types of interventions. |
| Schichtel M, Wee B, Perera R, Onakpoya I, Albury C. Effect of Behavior Change Techniques Targeting Clinicians to Improve Advance Care Planning in Heart Failure: A Systematic Review and Meta-Analysiset. *Ann Behav Med*. (2021) 55:383–398  doi: 10.1093/abm/kaaa075 | Targeted clinicians. |
| Scott-Sheldon L, Gathright E, Salmoirago-Blother E, Wu W-C. Women´s participation in stress management interventions for chronic heart failure: a meta-analysis of randomized controlled trials. *Psych, Health Med.* (2022) 27(4):761-779. doi: 10.1080/13548506.2021.1874436 | Did not report outcomes of the interventions. Reported women’s participation rates and predictors of participation in stress management interventions. |
