# Supplementary material for: A meta-review of systematic reviews and meta-analyses on outcomes of psychosocial interventions in heart failure
Source: Front Psychiatry. 2023 Mar 10;14:1095665. doi: 10.3389/fpsyt.2023.1095665 (PMC10036787; doi:10.3389/fpsyt.2023.1095665)
Supplement: Supplementary file 2 [file Table_2.DOCX]

Supplementary material 2. Overview of included original articles in the systematic reviews and meta-analyses.

|  | Systematic reviews and meta-analyses | | | | | | |
| --- | --- | --- | --- | --- | --- | --- | --- |
|  | Chernoff et al 2022 | Gathright et al 2021 | Helal et al 2020 | Jeyanantham et al 2017 | Jiang et al 2020 | Peng et al 2019 | Samartzis et al 2013 |
| Original articles (n=67) |  |  |  |  |  |  |  |
| Athilingam et al 2015 (1) |  |  |  |  |  | X |  |
| Austin et al 2005 (2) |  |  |  |  |  |  | X |
| Baker et al 2011 (3) |  |  |  |  | X |  |  |
| Barrow et al 2007 (4) |  | X |  |  |  |  |  |
| (Nahlén) Bose et al 2016 (5) |  | X |  |  | X |  |  |
| Brodie et al 2008 (6) |  |  |  |  | X |  |  |
| Cajanding 2016 (7) |  | X |  |  | X | X |  |
| Chang et al 2005 (8) |  | X |  |  |  |  | X |
| Chang et al 2016 (9) | X |  |  |  | X |  |  |
| Cline et al 1998 (10) |  |  |  |  |  |  | X |
| Creber et al 2016 (11) |  |  |  |  | X |  |  |
| Curiati et al 2005 (12) |  | X |  |  |  |  |  |
| Davis et al 2012 (13) |  |  |  |  | X |  |  |
| Dekker et al 2010* |  |  |  | X |  |  |  |
| Dekker et al 2011* |  |  |  | X |  |  |  |
| Dekker et al 2012 (14) | X | X | X | X | X | X |  |
| Doughty et al 2002 (15) |  |  |  |  |  |  | X |
| Dunbar et al 2015 (16) |  |  |  |  | X |  |  |
| Freedland et al 2015 (17) | X | X | X | X | X | X |  |
| Gary et al 2010 (18) | X | X | X | X | X | X | X |
| Goldberg et al 2003 (19) |  |  |  |  |  |  | X |
| Grady et al 2014 (20) |  |  |  |  | X |  |  |
| Harris et al 2019 (21) | X |  |  |  |  |  |  |
| Harrison et al 2002 (22) |  |  |  |  |  |  | X |
| Heo et al 2018 (23) |  |  | X |  |  |  |  |
| Huang et al 2016 (24) |  | X |  |  |  |  |  |
| Jaarsma et al 2000 (25) |  |  |  |  |  |  | X |
| Jayadevappa et al 2007 (26) | X | X |  |  | X |  |  |
| Kasper et al 2002 (27) |  |  |  |  |  |  | X |
| Khayati et al 2020 (28) | X |  |  |  |  |  |  |
| Krishna et al 2014 (29) |  | X |  |  |  |  |  |
| Lundgren et al 2015 (30) |  |  | X | X |  |  |  |
| Lundgren et al 2016 (31) | X |  | X |  |  | X |  |
| De Lusignan et al 2002 (32) |  |  |  |  |  |  | X |
| Luskin et al 2002 (33) | X |  |  |  |  |  | X |
| Naylor et al 2004 (34) |  |  |  |  |  |  | X |
| Paradis et al 2010 (35) |  |  |  |  | X |  |  |
| Pen 2016 (36) |  | X |  |  |  |  |  |
| Powell et al 2010 (37) |  | X |  |  | X |  |  |
| Pozehl et al 2010 (38) |  |  |  |  | X |  |  |
| Pressler et al 2015 (39) |  |  |  |  |  | X |  |
| Pullen et al 2008 (40) |  | X |  |  |  |  |  |
| Pullen et al 2010 (41) |  | X |  |  |  |  |  |
| Redeker et al 2015 (42) | X |  |  |  | X |  |  |
| Redeker et al 2017 (43) |  |  |  |  |  | X |  |
| Redwine et al 2019 (44) |  | X |  |  |  |  |  |
| Rich et al 1995 (45) |  |  |  |  |  |  | X |
| Riegel et al 2006 (46) |  |  |  |  |  |  | X |
| Seo et al 2016 (47) |  | X |  |  |  |  |  |
| Shao et al 2013 (48) |  |  |  |  | X |  |  |
| Shearer et al 2007 (49) |  |  |  |  | X |  |  |
| Sherwood et al 2017 (50) | X | X | X |  |  |  |  |
| Shively et al 2013 (51) |  |  |  |  | X |  |  |
| Smeulders et al 2010 (52) | X |  |  |  | X |  |  |
| Sullivan et al 2009 (53) |  |  | X |  |  |  |  |
| Swanson et al 2009 (54) | X | X |  |  |  |  |  |
| Tiede et al 2016 (55) |  |  |  |  | X |  |  |
| Tully et al 2015 (56) |  |  | X |  |  |  |  |
| Varma et al 1999 (57) |  |  |  |  |  |  | X |
| Wang et al 2014 (58) |  | X |  |  |  |  |  |
| Wang et al 2016 (59) |  |  |  |  | X |  |  |
| Wu et al 2012 (60) |  |  |  |  | X |  |  |
| Yeh et al 2004 (61) |  | X |  |  |  |  |  |
| Yeh et al 2011 (62) | X | X |  |  |  |  |  |
| Yu et al 2007 (63) | X | X |  |  | X |  |  |
| Yu et al 2010 (64) |  |  |  |  | X |  | X |
| Zamanzadeh et al. 2013 (65) |  |  |  |  | X |  |  |

* Personal communication. The Abstract: "Dekker Rl, Tovar Eg, Doering Lv, Bailey Al, Campbell Cl, Wright Jh, Bishop Ml, Moser Dk (2014) a Single Cognitive Behavior Therapy Session Improves Short-Term Depressive Symptoms in Hospitalized Patients with Heart Failure. Circulation 130: A13961" combined data for two separate RCTs; therefore, the lead author was contacted, and provided the individual patient data and corresponding protocols for the two studies referred to as Dekker (2010) and Dekker (2011).

1. Athilingam P, Edwards JD, Valdes EG, Ji M, Guglin M. Computerized Auditory Cognitive Training to Improve Cognition and Functional Outcomes in Patients with Heart Failure: Results of a Pilot Study. *Heart Lung* (2015) 44(2):120-8. Epub 20150112. doi: 10.1016/j.hrtlng.2014.12.004.

2. Austin J, Williams R, Ross L, Moseley L, Hutchison S. Randomised Controlled Trial of Cardiac Rehabilitation in Elderly Patients with Heart Failure. *Eur J Heart Fail* (2005) 7(3):411-7. doi: 10.1016/j.ejheart.2004.10.004.

3. Baker DW, Dewalt DA, Schillinger D, Hawk V, Ruo B, Bibbins-Domingo K, et al. The Effect of Progressive, Reinforcing Telephone Education and Counseling Versus Brief Educational Intervention on Knowledge, Self-Care Behaviors and Heart Failure Symptoms. *J Card Fail* (2011) 17(10):789-96. Epub 20110723. doi: 10.1016/j.cardfail.2011.06.374.

4. Barrow DE, Bedford A, Ives G, O'Toole L, Channer KS. An Evaluation of the Effects of Tai Chi Chuan and Chi Kung Training in Patients with Symptomatic Heart Failure: A Randomised Controlled Pilot Study. *Postgrad Med J* (2007) 83(985):717-21. doi: 10.1136/pgmj.2007.061267.

5. Nahlen Bose C, Persson H, Bjorling G, Ljunggren G, Elfstrom ML, Saboonchi F. Evaluation of a Coping Effectiveness Training Intervention in Patients with Chronic Heart Failure - a Randomized Controlled Trial. *Eur J Cardiovasc Nurs* (2016) 15(7):537-48. Epub 2016/01/07. doi: 10.1177/1474515115625033.

6. Brodie DA, Inoue A, Shaw DG. Motivational Interviewing to Change Quality of Life for People with Chronic Heart Failure: A Randomised Controlled Trial. *Int J Nurs Stud* (2008) 45(4):489-500. Epub 20070126. doi: 10.1016/j.ijnurstu.2006.11.009.

7. Cajanding RJ. The Effectiveness of a Nurse-Led Cognitive-Behavioral Therapy on the Quality of Life, Self-Esteem and Mood among Filipino Patients Living with Heart Failure: A Randomized Controlled Trial. *Appl Nurs Res* (2016) 31:86-93. Epub 20160120. doi: 10.1016/j.apnr.2016.01.002.

8. Chang BH, Hendricks A, Zhao Y, Rothendler JA, LoCastro JS, Slawsky MT. A Relaxation Response Randomized Trial on Patients with Chronic Heart Failure. *J Cardiopulm Rehabil* (2005) 25(3):149-57. Epub 2005/06/03.

9. Chang YL, Chiou AF, Cheng SM, Lin KC. Tailored Educational Supportive Care Programme on Sleep Quality and Psychological Distress in Patients with Heart Failure: A Randomised Controlled Trial. *Int J Nurs Stud* (2016) 61:219-29. Epub 20160704. doi: 10.1016/j.ijnurstu.2016.07.002.

10. Cline CM, Israelsson BY, Willenheimer RB, Broms K, Erhardt LR. Cost Effective Management Programme for Heart Failure Reduces Hospitalisation. *Heart* (1998) 80(5):442-6. doi: 10.1136/hrt.80.5.442.

11. Masterson Creber R, Patey M, Lee CS, Kuan A, Jurgens C, Riegel B. Motivational Interviewing to Improve Self-Care for Patients with Chronic Heart Failure: Miti-Hf Randomized Controlled Trial. *Patient Educ Couns* (2016) 99(2):256-64. Epub 20150829. doi: 10.1016/j.pec.2015.08.031.

12. Curiati JA, Bocchi E, Freire JO, Arantes AC, Braga M, Garcia Y, et al. Meditation Reduces Sympathetic Activation and Improves the Quality of Life in Elderly Patients with Optimally Treated Heart Failure: A Prospective Randomized Study. *J Altern Complement Med* (2005) 11(3):465-72. doi: 10.1089/acm.2005.11.465.

13. Davis KK, Mintzer M, Dennison Himmelfarb CR, Hayat MJ, Rotman S, Allen J. Targeted Intervention Improves Knowledge but Not Self-Care or Readmissions in Heart Failure Patients with Mild Cognitive Impairment. *Eur J Heart Fail* (2012) 14(9):1041-9. Epub 20120626. doi: 10.1093/eurjhf/hfs096.

14. Dekker RL, Moser DK, Peden AR, Lennie TA. Cognitive Therapy Improves Three-Month Outcomes in Hospitalized Patients with Heart Failure. *J Card Fail* (2012) 18(1):10-20. Epub 20111109. doi: 10.1016/j.cardfail.2011.09.008.

15. Doughty RN, Wright SP, Pearl A, Walsh HJ, Muncaster S, Whalley GA, et al. Randomized, Controlled Trial of Integrated Heart Failure Management: The Auckland Heart Failure Management Study. *Eur Heart J* (2002) 23(2):139-46. doi: 10.1053/euhj.2001.2712.

16. Dunbar SB, Reilly CM, Gary R, Higgins MK, Culler S, Butts B, et al. Randomized Clinical Trial of an Integrated Self-Care Intervention for Persons with Heart Failure and Diabetes: Quality of Life and Physical Functioning Outcomes. *J Card Fail* (2015) 21(9):719-29. Epub 20150529. doi: 10.1016/j.cardfail.2015.05.012.

17. Freedland KE, Carney RM, Rich MW, Steinmeyer BC, Rubin EH. Cognitive Behavior Therapy for Depression and Self-Care in Heart Failure Patients: A Randomized Clinical Trial. *JAMA Intern Med* (2015) 175(11):1773-82. doi: 10.1001/jamainternmed.2015.5220.

18. Gary RA, Dunbar SB, Higgins MK, Musselman DL, Smith AL. Combined Exercise and Cognitive Behavioral Therapy Improves Outcomes in Patients with Heart Failure. *J Psychosom Res* (2010) 69(2):119-31. Epub 20100312. doi: 10.1016/j.jpsychores.2010.01.013.

19. Goldberg LR, Piette JD, Walsh MN, Frank TA, Jaski BE, Smith AL, et al. Randomized Trial of a Daily Electronic Home Monitoring System in Patients with Advanced Heart Failure: The Weight Monitoring in Heart Failure (Wharf) Trial. *Am Heart J* (2003) 146(4):705-12. doi: 10.1016/s0002-8703(03)00393-4.

20. Grady KL, de Leon CF, Kozak AT, Cursio JF, Richardson D, Avery E, et al. Does Self-Management Counseling in Patients with Heart Failure Improve Quality of Life? Findings from the Heart Failure Adherence and Retention Trial (Hart). *Qual Life Res* (2014) 23(1):31-8. Epub 20130607. doi: 10.1007/s11136-013-0432-7.

21. Harris KM, Schiele SE, Emery CF. Pilot Randomized Trial of Brief Behavioral Treatment for Insomnia in Patients with Heart Failure. *Heart Lung* (2019) 48(5):373-80. Epub 20190627. doi: 10.1016/j.hrtlng.2019.06.003.

22. Harrison MB, Browne GB, Roberts J, Tugwell P, Gafni A, Graham ID. Quality of Life of Individuals with Heart Failure: A Randomized Trial of the Effectiveness of Two Models of Hospital-to-Home Transition. *Med Care* (2002) 40(4):271-82. doi: 10.1097/00005650-200204000-00003.

23. Heo S, McSweeney J, Ounpraseuth S, Shaw-Devine A, Fier A, Moser DK. Testing a Holistic Meditation Intervention to Address Psychosocial Distress in Patients with Heart Failure: A Pilot Study. *J Cardiovasc Nurs* (2018) 33(2):126-34. doi: 10.1097/jcn.0000000000000435.

24. Huang T-Y, Moser DK, Hwang S-L. The Short-Term and Long-Term Effects of Biofeedback-Assisted Relaxation Therapy in Patients with Heart Failure:A Randomized Control Study. *SAGE Open Nursing* (2016) 2:2377960816680825. doi: 10.1177/2377960816680825.

25. Jaarsma T, Halfens R, Tan F, Abu-Saad HH, Dracup K, Diederiks J. Self-Care and Quality of Life in Patients with Advanced Heart Failure: The Effect of a Supportive Educational Intervention. *Heart Lung* (2000) 29(5):319-30. doi: 10.1067/mhl.2000.108323.

26. Jayadevappa R, Johnson JC, Bloom BS, Nidich S, Desai S, Chhatre S, et al. Effectiveness of Transcendental Meditation on Functional Capacity and Quality of Life of African Americans with Congestive Heart Failure: A Randomized Control Study. *Ethn Dis* (2007) 17(1):72-7.

27. Kasper EK, Gerstenblith G, Hefter G, Van Anden E, Brinker JA, Thiemann DR, et al. A Randomized Trial of the Efficacy of Multidisciplinary Care in Heart Failure Outpatients at High Risk of Hospital Readmission. *J Am Coll Cardiol* (2002) 39(3):471-80. doi: 10.1016/s0735-1097(01)01761-2.

28. Khayati R, Rezaee N, Shakiba M, Navidian A. The Effect of Cognitive-Behavioral Training Versus Conventional Training on Self-Care and Depression Severity in Heart Failure Patients with Depression: A Randomized Clinical Trial. *J Caring Sci* (2020) 9(4):203-11. Epub 20201201. doi: 10.34172/jcs.2020.31.

29. Krishna BH, Pal P, Pal G, Balachander J, Jayasettiaseelon E, Sreekanth Y, et al. A Randomized Controlled Trial to Study the Effect of Yoga Therapy on Cardiac Function and N Terminal Pro Bnp in Heart Failure. *Integr Med Insights* (2014) 9:1-6. Epub 20140401. doi: 10.4137/imi.S13939.

30. Lundgren J, Andersson G, Dahlstrom O, Jaarsma T, Kohler AK, Johansson P. Internet-Based Cognitive Behavior Therapy for Patients with Heart Failure and Depressive Symptoms: A Proof of Concept Study. *Patient Educ Couns* (2015) 98(8):935-42. Epub 2015/05/21. doi: 10.1016/j.pec.2015.04.013.

31. Lundgren JG, Dahlstrom O, Andersson G, Jaarsma T, Karner Kohler A, Johansson P. The Effect of Guided Web-Based Cognitive Behavioral Therapy on Patients with Depressive Symptoms and Heart Failure: A Pilot Randomized Controlled Trial. *J Med Internet Res* (2016) 18(8):e194. Epub 2016/08/05. doi: 10.2196/jmir.5556.

32. de Lusignan S, Wells S, Johnson P, Meredith K, Leatham E. Compliance and Effectiveness of 1 Year's Home Telemonitoring. The Report of a Pilot Study of Patients with Chronic Heart Failure. *Eur J Heart Fail* (2001) 3(6):723-30. doi: 10.1016/s1388-9842(01)00190-8.

33. Luskin F, Reitz M, Newell K, Quinn TG, Haskell W. A Controlled Pilot Study of Stress Management Training of Elderly Patients with Congestive Heart Failure. *Prev Cardiol* (2002) 5(4):168-72. Epub 2002/11/06.

34. Naylor MD, Brooten DA, Campbell RL, Maislin G, McCauley KM, Schwartz JS. Transitional Care of Older Adults Hospitalized with Heart Failure: A Randomized, Controlled Trial. *J Am Geriatr Soc* (2004) 52(5):675-84. doi: 10.1111/j.1532-5415.2004.52202.x.

35. Paradis V, Cossette S, Frasure-Smith N, Heppell S, Guertin MC. The Efficacy of a Motivational Nursing Intervention Based on the Stages of Change on Self-Care in Heart Failure Patients. *J Cardiovasc Nurs* (2010) 25(2):130-41. doi: 10.1097/JCN.0b013e3181c52497.

36. Pen XF. Effect of Taijiquan Exercise on Cardiac Function and Quality of Life in Patients with Chronic Heart Failure. *Chinese Journal of Physical Medicine and Rehabilitation* (2016) (38):51-3.

37. Powell LH, Calvin JE, Jr., Richardson D, Janssen I, Mendes de Leon CF, Flynn KJ, et al. Self-Management Counseling in Patients with Heart Failure: The Heart Failure Adherence and Retention Randomized Behavioral Trial. *JAMA* (2010) 304(12):1331-8. doi: 10.1001/jama.2010.1362.

38. Pozehl B, Duncan K, Hertzog M, Norman JF. Heart Failure Exercise and Training Camp: Effects of a Multicomponent Exercise Training Intervention in Patients with Heart Failure. *Heart Lung* (2010) 39(6 Suppl):S1-13. Epub 20100703. doi: 10.1016/j.hrtlng.2010.04.008.

39. Pressler SJ, Titler M, Koelling TM, Riley PL, Jung M, Hoyland-Domenico L, et al. Nurse-Enhanced Computerized Cognitive Training Increases Serum Brain-Derived Neurotropic Factor Levels and Improves Working Memory in Heart Failure. *J Card Fail* (2015) 21(8):630-41. Epub 20150514. doi: 10.1016/j.cardfail.2015.05.004.

40. Pullen PR, Nagamia SH, Mehta PK, Thompson WR, Benardot D, Hammoud R, et al. Effects of Yoga on Inflammation and Exercise Capacity in Patients with Chronic Heart Failure. *J Card Fail* (2008) 14(5):407-13. Epub 20080527. doi: 10.1016/j.cardfail.2007.12.007.

41. Pullen PR, Thompson WR, Benardot D, Brandon LJ, Mehta PK, Rifai L, et al. Benefits of Yoga for African American Heart Failure Patients. *Med Sci Sports Exerc* (2010) 42(4):651-7. doi: 10.1249/MSS.0b013e3181bf24c4.

42. Redeker NS, Jeon S, Andrews L, Cline J, Jacoby D, Mohsenin V. Feasibility and Efficacy of a Self-Management Intervention for Insomnia in Stable Heart Failure. *J Clin Sleep Med* (2015) 11(10):1109-19. Epub 20151015. doi: 10.5664/jcsm.5082.

43. Redeker NS, Jeon S, Andrews L, Cline J, Mohsenin V, Jacoby D. Effects of Cognitive Behavioral Therapy for Insomnia on Sleep-Related Cognitions among Patients with Stable Heart Failure. *Behav Sleep Med* (2019) 17(3):342-54. Epub 20170822. doi: 10.1080/15402002.2017.1357120.

44. Redwine LS, Wilson K, Pung MA, Chinh K, Rutledge T, Mills PJ, et al. A Randomized Study Examining the Effects of Mild-to-Moderate Group Exercises on Cardiovascular, Physical, and Psychological Well-Being in Patients with Heart Failure. *J Cardiopulm Rehabil Prev* (2019) 39(6):403-8. doi: 10.1097/hcr.0000000000000430.

45. Rich MW, Beckham V, Wittenberg C, Leven CL, Freedland KE, Carney RM. A Multidisciplinary Intervention to Prevent the Readmission of Elderly Patients with Congestive Heart Failure. *N Engl J Med* (1995) 333(18):1190-5. doi: 10.1056/nejm199511023331806.

46. Riegel B, Carlson B, Glaser D, Romero T. Randomized Controlled Trial of Telephone Case Management in Hispanics of Mexican Origin with Heart Failure. *J Card Fail* (2006) 12(3):211-9. doi: 10.1016/j.cardfail.2006.01.005.

47. Seo Y, Yates B, LaFramboise L, Pozehl B, Norman JF, Hertzog M. A Home-Based Diaphragmatic Breathing Retraining in Rural Patients with Heart Failure. *West J Nurs Res* (2016) 38(3):270-91. Epub 20150508. doi: 10.1177/0193945915584201.

48. Shao JH, Chang AM, Edwards H, Shyu YI, Chen SH. A Randomized Controlled Trial of Self-Management Programme Improves Health-Related Outcomes of Older People with Heart Failure. *J Adv Nurs* (2013) 69(11):2458-69. Epub 20130312. doi: 10.1111/jan.12121.

49. Shearer NB, Cisar N, Greenberg EA. A Telephone-Delivered Empowerment Intervention with Patients Diagnosed with Heart Failure. *Heart Lung* (2007) 36(3):159-69. doi: 10.1016/j.hrtlng.2006.08.006.

50. Sherwood A, Blumenthal JA, Koch GG, Hoffman BM, Watkins LL, Smith PJ, et al. Effects of Coping Skills Training on Quality of Life, Disease Biomarkers, and Clinical Outcomes in Patients with Heart Failure: A Randomized Clinical Trial. *Circ Heart Fail* (2017) 10(1). Epub 2017/01/08. doi: 10.1161/circheartfailure.116.003410.

51. Shively MJ, Gardetto NJ, Kodiath MF, Kelly A, Smith TL, Stepnowsky C, et al. Effect of Patient Activation on Self-Management in Patients with Heart Failure. *J Cardiovasc Nurs* (2013) 28(1):20-34. doi: 10.1097/JCN.0b013e318239f9f9.

52. Smeulders ES, van Haastregt JC, Ambergen T, Uszko-Lencer NH, Janssen-Boyne JJ, Gorgels AP, et al. Nurse-Led Self-Management Group Programme for Patients with Congestive Heart Failure: Randomized Controlled Trial. *Journal of Advanced Nursing* (2010) 66(7):1487-99. Epub 2010/05/25. doi: 10.1111/j.1365-2648.2010.05318.x.

53. Sullivan MJ, Wood L, Terry J, Brantley J, Charles A, McGee V, et al. The Support, Education, and Research in Chronic Heart Failure Study (Search): A Mindfulness-Based Psychoeducational Intervention Improves Depression and Clinical Symptoms in Patients with Chronic Heart Failure. *Am Heart J* (2009) 157(1):84-90. Epub 2008/12/17. doi:10.1016/j.ahj.2008.08.033.

54. Swanson KS, Gevirtz RN, Brown M, Spira J, Guarneri E, Stoletniy L. The Effect of Biofeedback on Function in Patients with Heart Failure. *Appl Psychophysiol Biofeedback* (2009) 34(2):71-91. Epub 20090210. doi: 10.1007/s10484-009-9077-2.

55. Tiede M, Dwinger S, Herbarth L, Härter M, Dirmaier J. Long-Term Effectiveness of Telephone-Based Health Coaching for Heart Failure Patients: A Post-Only Randomised Controlled Trial. *J Telemed Telecare* (2017) 23(8):716-24. Epub 20160907. doi: 10.1177/1357633x16668436.

56. Tully PJ, Selkow T, Bengel J, Rafanelli C. A Dynamic View of Comorbid Depression and Generalized Anxiety Disorder Symptom Change in Chronic Heart Failure: The Discrete Effects of Cognitive Behavioral Therapy, Exercise, and Psychotropic Medication. *Disabil Rehabil* (2015) 37(7):585-92. Epub 20140701. doi: 10.3109/09638288.2014.935493.

57. Varma S, McElnay JC, Hughes CM, Passmore AP, Varma M. Pharmaceutical Care of Patients with Congestive Heart Failure: Interventions and Outcomes. *Pharmacotherapy* (1999) 19(7):860-9. doi: 10.1592/phco.19.10.860.31565.

58. Wang LN, Tao H, Zhao Y, Zhou YQ, Jiang XR. Optimal Timing for Initiation of Biofeedback-Assisted Relaxation Training in Hospitalized Coronary Heart Disease Patients with Sleep Disturbances. *J Cardiovasc Nurs* (2014) 29(4):367-76. doi: 10.1097/JCN.0b013e318297c41b.

59. Wang TC, Huang JL, Ho WC, Chiou AF. Effects of a Supportive Educational Nursing Care Programme on Fatigue and Quality of Life in Patients with Heart Failure: A Randomised Controlled Trial. *Eur J Cardiovasc Nurs* (2016) 15(2):157-67. Epub 20151119. doi: 10.1177/1474515115618567.

60. Wu JR, Corley DJ, Lennie TA, Moser DK. Effect of a Medication-Taking Behavior Feedback Theory-Based Intervention on Outcomes in Patients with Heart Failure. *J Card Fail* (2012) 18(1):1-9. Epub 20111019. doi: 10.1016/j.cardfail.2011.09.006.

61. Yeh GY, Wood MJ, Lorell BH, Stevenson LW, Eisenberg DM, Wayne PM, et al. Effects of Tai Chi Mind-Body Movement Therapy on Functional Status and Exercise Capacity in Patients with Chronic Heart Failure: A Randomized Controlled Trial. *Am J Med* (2004) 117(8):541-8. doi: 10.1016/j.amjmed.2004.04.016.

62. Yeh GY, McCarthy EP, Wayne PM, Stevenson LW, Wood MJ, Forman D, et al. Tai Chi Exercise in Patients with Chronic Heart Failure: A Randomized Clinical Trial. *Arch Intern Med* (2011) 171(8):750-7. doi: 10.1001/archinternmed.2011.150.

63. Yu DS, Lee DT, Woo J. Effects of Relaxation Therapy on Psychologic Distress and Symptom Status in Older Chinese Patients with Heart Failure. *J Psychosom Res* (2007) 62(4):427-37. doi: 10.1016/j.jpsychores.2006.10.012.

64. Yu DSF, Lee DTF, Woo J. Improving Health-Related Quality of Life of Patients with Chronic Heart Failure: Effects of Relaxation Therapy. *Journal of Advanced Nursing* (2010) 66(2):392-403. doi: 10.1111/j.1365-2648.2009.05198.x.

65. Zamanzadeh V, Valizadeh L, Howard AF, Jamshidi F. A Supportive-Educational Intervention for Heart Failure Patients in Iran: The Effect on Self-Care Behaviours. *Nurs Res Pract* (2013) 2013:492729. Epub 20130922. doi: 10.1155/2013/492729.
